# An efficient and reliable method for measuring cerebral lateralization during speech with functional transcranial Doppler ultrasound

**DOI:** 10.1016/j.neuropsychologia.2008.09.013

**Published:** 2009-01

**Authors:** Dorothy V.M. Bishop, Helen Watt, Marietta Papadatou-Pastou

**Affiliations:** University of Oxford, United Kingdom

**Keywords:** Cerebral lateralization, Child, Functional transcranial Doppler, Language, Speech

## Abstract

The gold standard method for measuring cerebral lateralization, the Wada technique, is too invasive for routine research use. Functional magnetic resonance imaging is a viable alternative but it is costly and affected by muscle artefact when activation tasks involve speech. Functional transcranial Doppler ultrasonography (fTCD) can be used to assess cerebral lateralization by comparing blood flow in the middle cerebral arteries. We used fTCD to compare indices of language lateralization in 33 adults in three different paradigms: Word Generation, Picture Description and a shorter Animation Description task. Animation Description gave valid results, and we subsequently demonstrated its reliability in a group of 21 4-year-old children. Cerebral lateralization during spoken language generation can be assessed reliably and cheaply using fTCD with a paradigm that is less taxing than the traditional word generation paradigm, does not require literacy skills and can be completed in 15 min or less.

## 1 Introduction

For many years, the only way of reliably assessing cerebral lateralization for speech in individuals was the Wada technique, an invasive method in which function of one cerebral hemisphere was transiently disrupted by administration of sodium amytal via a carotid artery (Wada & Rasmussen, 1960). This method has been widely used in presurgical assessment of patients with epilepsy, to establish which hemisphere is dominant for language, but is not feasible for studies with non-clinical samples. Functional magnetic resonance imaging (fMRI) is starting to replace the Wada technique in clinical assessment, but is too expensive for routine use in research studies (Pelletier, Sauerwein, Lepore, Saint-Amour, & Lassonde, 2007). Functional transcranial Doppler ultrasound (fTCD) has been shown to be a reliable alternative that is both non-invasive and inexpensive (Deppe, Ringelstein, & Knecht, 2004). This method uses ultrasound to measure event-related changes in blood flow in the middle cerebral arteries (MCA). A breakthrough in the use of fTCD for this purpose came from the work of Deppe and colleagues, who devised analytic methods that took into account both the activity from the heart rate cycle, and any differences in overall blood flow between left and right sides, using an analysis package, ‘Average’ (Deppe, Knecht, Henningsen, & Ringelstein, 1997). Prior to this, measurements of blood flow in left and right MCAs tended to be too noisy to give reliable results. However, with these more sophisticated techniques, it was possible to detect perfusion asymmetries between the two MCAs of around 1%, and to show reliable left hemisphere activation for speech-based tasks in typical adults. The method gives high correlations with both the Wada technique (Knecht et al., 1998) and fMRI measures of cerebral lateralization (Deppe et al., 2000).

The gold standard method used to demonstrate cerebral lateralization for speech is the word generation (WG) task, which is particularly effective in demonstrating lateralization in fMRI studies (Benson et al., 1999). The participant is shown a letter and asked to silently generate as many words as possible beginning with that letter. This task allows the study of brain correlates of language generation in the absence of overt speech, thus avoiding any artefact associated with movement of the articulators. After an interval of silent word generation, the participant may be asked to speak the words, to confirm that words were generated, before having a rest period to allow task-related activation to return to baseline. This method has been adopted with fTCD, with a laterality index (LI) computed by averaging blood flow difference for 20 or more trials of this kind, for a 2 s period centred on the maximal left–right difference during the word generation interval.

A limitation of the WG task is that it is not suitable for participants with poor literacy skills, including children. Furthermore, many participants find the task quite taxing, and the inclusion of a 35 s rest period means that each trial lasts 1 min, giving an overall presentation time of over 20 min. For robust adults without neurological problems this is not a problem, but for assessment of children and clinical patients, a less demanding paradigm would be desirable. Lohmann, Drager, Muller-Ehrenberg, Deppe, and Knecht (2005) developed an alternative Picture Description task that was suitable for younger participants, in which each epoch consisted of presentation of a coloured picture of an object, e.g., an apple, and the participant was asked to say as much as they could about the object. Although this task involved overt speech, cerebral lateralization was still observed, and in a sample of 9 adults and 3 literate children good agreement was observed between this method and the WG task. This suggested that, in contrast to fMRI, in fTCD the motor movements of overt speech may not mask the language-specific activation. Lohmann et al. demonstrated good reliability of the method in a sample of 16 children aged 2–9 years, but they noted that their sample had a selection bias in favour of linguistically proficient children. The task involved 1-min trials, making total duration for 30 trials of half an hour, and requiring lengthy rest periods where the participant is required to sit quietly, raising questions regarding its feasibility with less able or distractible children.

The current study was designed to compare the sensitivity of different fTCD paradigms for assessing cerebral lateralization for speech, with the goal of evaluating a novel activation task designed to be more engaging for children. This was similar to the Picture Description task, except that in each epoch, participants viewed a clip of a cartoon, and then were asked to describe what had happened after the animation ended. This task used a shorter interval between stimulus presentations than was used for the other two methods, and used the period of viewing the cartoon as a baseline period, rather than requiring a prolonged period of sitting doing nothing.

## 2 Methods

### 2.1 Participants

Adult participants (aged 20–64) were recruited from Oxford University staff and students, and their acquaintances. Preference was given to left-handers in recruitment, with the aim of achieving a range of cerebral lateralization. Handedness was assessed using the Edinburgh Handedness Inventory (Oldfield, 1971), but for the purposes of analysis here a simple division was made on the basis of writing hand between right-handers (N = 21) and left-handers (N = 12). Two further potential participants were seen but did not produce useable data because a signal could not be obtained from the MCA (one case), or was too noisy (one case).

Child participants were 21 typically developing 4-year-olds (12 male and 9 female, mean age 4.08 years, SD .06 years) who were taking part in a longitudinal study of language development. Handedness was assessed using a handedness inventory suitable for young children. Handedness was not a selection criterion, but all children preferred the right hand for using a pencil. A further four children were dropped from the study because of failure to talk during the description phase (one case), technical failure (one case) or noisy recordings (two cases).

All adult participants gave written informed consent, whereas parental consent and child assent were obtained for the 4-year-old children. The project was approved by the Central University Research Ethics Committee of the University of Oxford.

### 2.2 Apparatus

Bilateral blood flow was measured using a commercially available Doppler ultrasonography device (DWL Multidop T2: manufacturer, DWL Elektronische Systeme, Singen, Germany), using two 2-MHz transducer probes mounted on a flexible headset. Visual stimuli (letters, pictures and video clips) were presented on a PC controlled by Presentation software (Neurobehavioral systems), which sent marker pulses to the Multidop system to mark the start of each epoch.

### 2.3 Stimuli

The *WG* paradigm was presented as described by Knecht et al. (1998). A total of 23 trials was presented, with a 1 min duration for each trial. In each trial there was an initial 5 s interval during which a ‘clear mind’ message was displayed on the screen, then a 15 s interval during which the participant silently generated words to match a letter displayed on the screen. The participant then spoke the generated words during a 5 s period, and had instructions to rest for the remaining 35 s of the trial. Of the 26 letters Q, X and Z were omitted and the remaining letters were displayed in random order.

The *Picture Description* paradigm was as described by Lohmann et al. (2005). Thirty trials were presented with a 1-min duration for each trial. The sequence of events was similar to the WG paradigm, except that the stimulus was a picture of a common object (e.g., apple, bike), and the participant started describing the picture as soon as it was displayed. The final rest period was initiated 30 s after the onset of the picture and lasted 25 s.

The *Animation Description* paradigm used 12 s clips from a children’s cartoon.^1^ The cartoon included sounds but no speech. Thirty clips were used, with a 30 s duration for each trial. We had previously established in pilot studies that there was no evidence of lateralised activation while participants passively watched these video clips. For the current study, while each clip was presented, the participant was asked to remain silent, and then to describe what had been seen when an auditory signal was presented to coincide with the end of the clip. The picture description period lasted 10 s, with the final 8 s being a silent rest period. Note that whereas for the other two tasks, during the baseline period before the start of the epoch the participant merely watches a screen instructing them to relax or to prepare to see the stimulus, during this task, the participant watches the animation during the pre-speaking baseline period.

### 2.4 Procedure

All adult participants completed all three activation paradigms in one or two sessions. Children completed only the Animation Description paradigm.

### 2.5 Data analysis

**Fig. 1:**
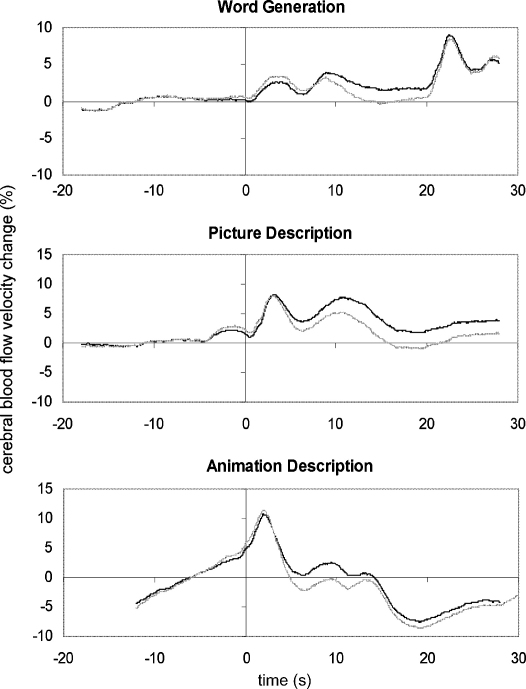
Adults: average activation across epoch for left (black) and right (grey) MCA.

Data from each fTCD paradigm were analysed using the Average software, with the data being processed using the Autoedit function of Average 1.85, which downsamples the blood flow envelope from each probe to 25 Hz, adds a channel corresponding to the heart beat, normalises the left and right cerebral blood flow velocity curve to a mean of 100%, and removes heart beat activity, using the heart cycle integration described by Deppe et al. (1997). Time-locked epochs are then averaged, after rejecting epochs with unusually high or low levels of activity. The mean difference curve for left and right channels was corrected to give a mean value of zero over a baseline period of 12 s prior to the presentation of the stimulus. A longer baseline has been used in prior studies with WG and Picture Description, but the faster presentation rate of the Animation task allowed only for a 12 s baseline, so this was used for all tasks for comparability. Periods of interest were based on previous research for the WG and Picture Description tasks (8–18 s after letter onset for WG, and 13–30 s after picture onset for Picture Description). For the Animation Description task the period of interest was defined as 4–14 s after onset of the cue to speak, based on inspection of the grand average means (Fig. 1). The LI is defined as the mean cerebral blood flow velocity change in a 2 s window centred on the peak value in the period of interest. A positive LI indicates greater left than right hemisphere activation. For each paradigm, split-half reliability was assessed by computing the LI for odd and even epochs.

## 3 Results

### 3.1 Adults

Mean activation plots for adults, averaged over all epochs, in the three paradigms are shown in Fig. 1. Table 1 shows the mean LIs for all three tasks, which differed significantly from zero in all cases.

Split-half reliabilities were computed by computing the LI values for odd and even epochs, and correlating these. Reliabilities were .89, .93 and .91 for the WG, Picture Description and Animation Description tasks respectively.

Correlations between the LIs computed from the three tasks are shown in Table 2. Because data were skewed, with only a handful of participants showing right hemisphere speech, Spearman correlations are presented to indicate the level of correlation for the ranked data; this minimizes the effect of outliers. Correlations are strongest for the two tasks that involved speech, i.e., Picture Description and Animation Description.

Mean LI values were closely similar for left- and right-handers in this sample for all of the tasks: t-values were 0.61, −0.14 and −0.39 for the WG, Picture Description and Animation Description tasks respectively. As well as computing a LI, it is possible to categorise a person as having left-, bilateral or right-hemisphere language, using the standard error of the LI across epochs to determine if the LI is significantly different from zero. On this criterion, 15% were right-lateralised and 3% bilateral on the WG task, 6% were right-lateralised and 12% bilateral on Picture Description, and 15% right-lateralised and 3% bilateral on Animation Description. In no paradigm was there a suggestion of a difference in frequency for right- and left-handers.

### 3.2 Four-year-old children

The mean number of Animation Description epochs included in the LI computation was 25.1 (SD = 6.3, range 11–30). The mean LI was 1.90 (SD = 3.82, range −8.6 to 7.3), which is significantly different from zero on a t-test (t = 2.28, d.f. = 20, p = .034). The odd–even split-half reliability was .88. When considered categorically, four cases (19%) were right-lateralised and four (19%) were bilateral. This is comparable to the proportions observed in the child sample of Lohmann et al. (2005), where 19% were right-lateralised and 12% were bilateral, with all of these cases being right-handed.

## 4 Discussion

This study confirms that fTCD provides a useful method for assessing cerebral lateralization in situations where more expensive or invasive methods are not practical. The split-half reliabilities were high for all three tasks, indicating that one can get a reliable estimate of cerebral lateralization using 20–30 epochs. The two methods that are suitable for non-literate participants, Picture Description and Animation Description, gave good agreement, even though the epoch length was halved in the animation task, giving a much shorter resting interval. Furthermore the Animation Description task showed good split-half reliability in children. This result is encouraging in showing that fTCD can be used to assess cerebral lateralization in a pediatric population. It appeared as valid and reliable as the other methods, despite using a faster presentation rate, with a baseline period where the participant watched the animation.

The correlation between LIs from different tasks was highly significant but lower than the split-half reliability of individual tasks. This should perhaps not surprise us, given the different linguistic and cognitive operations involved in the tasks: Word Generation places heavy demands on phonological fluency, Picture Description also taxes generativity as well as semantics and syntax, and Animation Description places heaviest demands on sentence production. Different regions in the MCA territory will be implicated in each task, and it seems feasible that extent of cerebral lateralization may show task-specific variation. Because of the poor spatial resolution of fTCD it is not well-suited to investigate such effects, though it could be used to generate predictions about individual differences in task-specific laterality that could be investigated using fMRI.

The lack of relationship between handedness and cerebral lateralization was unexpected, but could be a consequence of lack of power, bearing in mind that data from other sources gives estimates of around 95% left-hemisphere speech in right-handers, and 67% left-hemisphere speech in left-handers (Bishop, 1990). Knecht, Deppe, et al. (2000) and Knecht, Draeger, et al. (2000) did find a relationship between handedness and cerebral lateralization on the WG task, but they used a much larger sample: 326 healthy adults, just under half of whom were left- or mixed-handers.

In conclusion, reliable assessment of cerebral lateralization with fTCD can be achieved using tasks that involve overt speech production. This method is already recognised as attractive in terms of low cost, portability and non-invasiveness; these results indicate that it also has benefits in terms of being applicable to individuals who are unable to do silent language generation tasks. Although we would recommend using as many trials as feasible, the high split-half reliability of the Animation Description task, suggests that a valid indication of cerebral lateralization can be obtained with this task using as few as fifteen 30-s trials.

## Figures and Tables

**Table 1:** Mean Laterality Indices (% cerebral blood flow velocity), with t-value for testing difference from zero.

	N	Mean	Range	SD	t-Value	p
Word generation	33	1.69	−5.11 to 5.05	2.53	3.85	.001
Picture description	33	3.28	−5.45 to 7.81	3.17	5.94	<.001
Animation description	33	2.55	−4.67 to 7.07	2.98	4.91	<.001

**Table 2:** Pearson (upper diagonal) and Spearman (lower diagonal) correlations between laterality indices; all correlations were significant at .01 level.

	WG LI	Pic LI	Anim LI
WG LI	1.00	.73	.68
Pic LI	.51	1.00	.70
Anim LI	.47	.62	1.00

## Footnotes

1 The .avi files for animations are available from the first author on request.
